# Supplementary material for: Surgical, Histopathological, and Quality of Life Outcomes Following Neoadjuvant Chemotherapy and Pancreatectomy for Borderline Resectable and Locally Advanced Pancreatic Cancer
Source: Cancers (Basel). 2025 Jul 29;17(15):2505. doi: 10.3390/cancers17152505 (PMC12346083; doi:10.3390/cancers17152505)
Supplement: Supplementary file 1 [file cancers-17-02505-s001.zip › cancers-3695309-supplementary.pdf]

Surgical, Histopathological, and Quality of Life Outcomes Following Neoadjuvant Chemotherapy and Pancreatectomy for Borderline Resectable and Locally Advanced Pancreatic Cancer

Ingvild Farnes [1,2], Caroline S. Verbeke [2,3], Dyre Kleive [1], Anne Waage [1], Tore Tholfsen [1], Milada Hagen [4], Bjarte Fosby [5], Pål-Dag Line [2,5] and Knut Jørgen Labori [1,2,*]

[1] Department of Hepato-Pancreato-Biliary Surgery, Oslo University Hospital, Rikshospitalet, 0372 Oslo, Norway; infarn@ous-hf.no (I.F.); dyrkle@ous-hf.no (D.K.); uxawaa@ous-hf.no (A.W.); tortho@ous-hf.no (T.T.)

[2] Institute of Clinical Medicine, University of Oslo, 0313 Oslo, Norway; c.s.verbeke@medisin.uio.no (C.S.V.); p.d.line@medisin.uio.no (P.-D.L.)

[3] Department of Pathology, Oslo University Hospital, Rikshospitalet, 0450 Oslo, Norway

[4] Department of Health Science and Biostatistics, Oslo Metropolitan University, 0130 Oslo, Norway; milasm@oslomet.no

[5] Department of Transplantation Medicine, Oslo University Hospital, 0372 Oslo, Norway; bfosby@ous-hf.no

* Correspondence: k.j.labori@medisin.uio.no; Tel.: +47-23070000

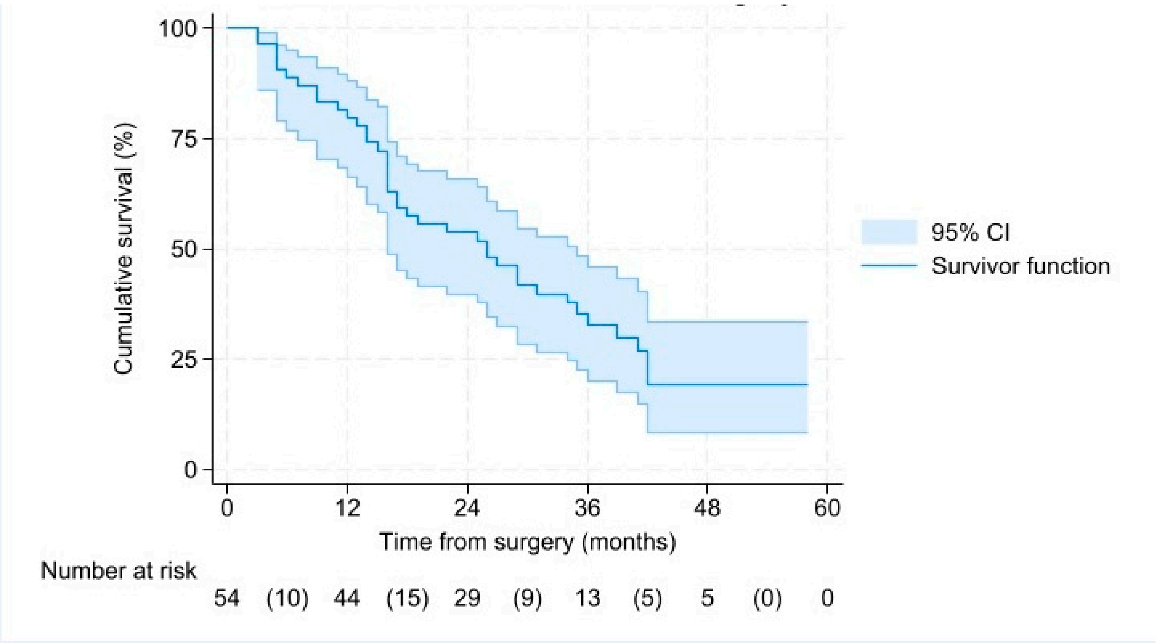

Supplementary Figure S1. Overall survival from time of surgery.

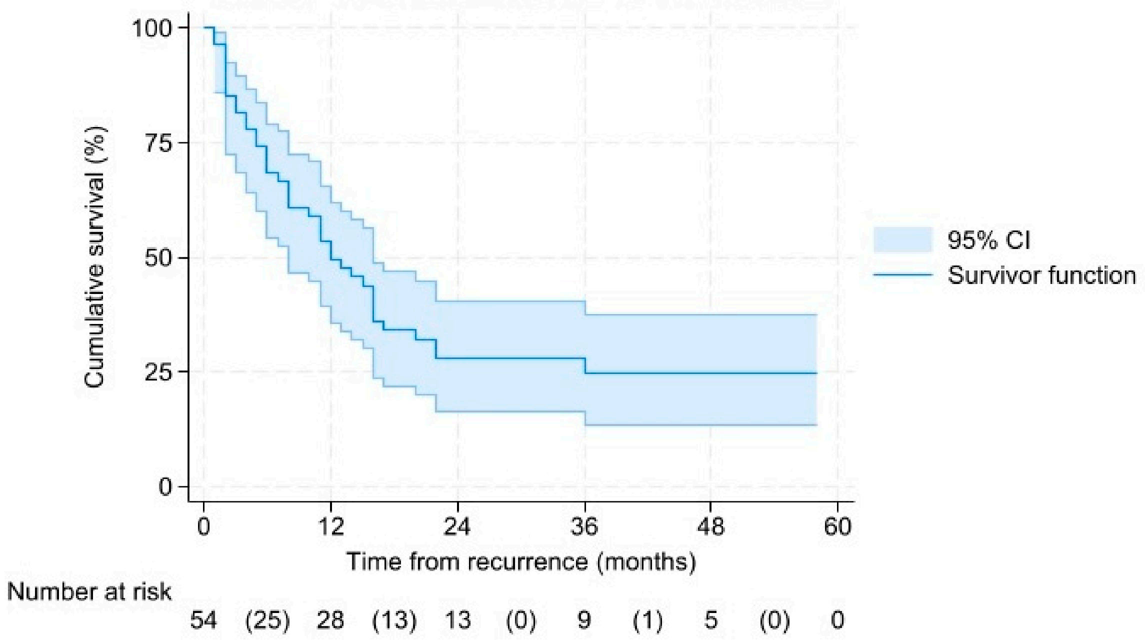

Supplementary Figure S2. Overall survival from time of recurrence.

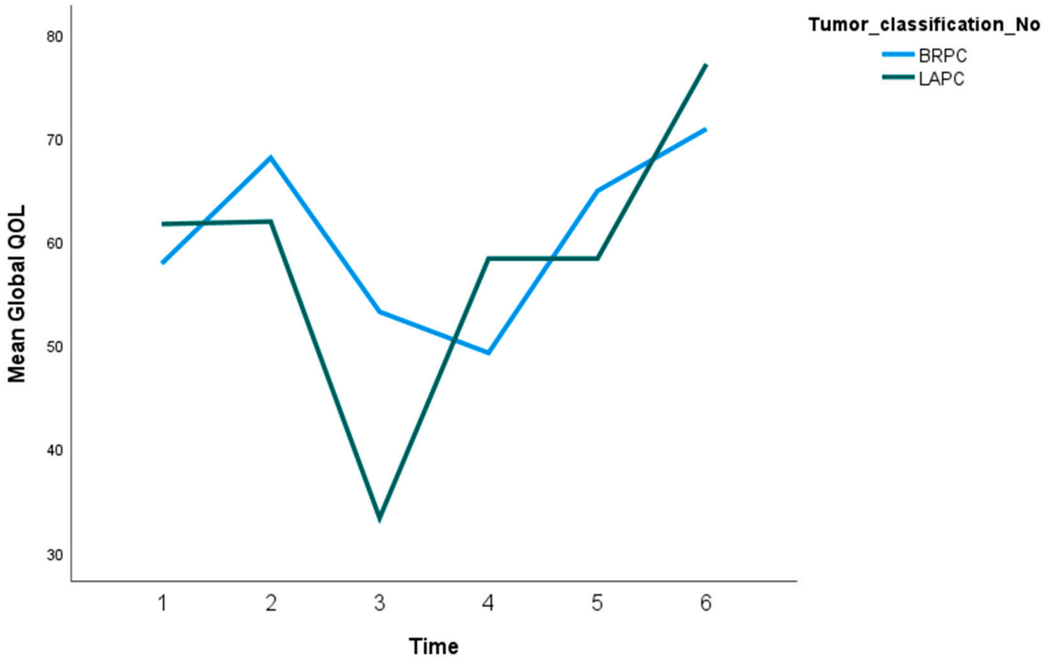

Supplementary Figure S3. Global Quality of Life of EORTC QLQ C-30. Higher scores indicate better functioning.

Time points: 1: baseline, 2: before surgery after completion of neoadjuvant chemotherapy, 3: 6 weeks postoperatively, 4: 3 months postoperatively, 5: 6 months postoperatively, 6: 12 months postoperatively

Supplementary Table S1. Major complications.

Clavien Dindo grade	Type of complication	Intervention
3a	Intraabdominal abscess	Percutaneous drainage
3a	Intraabdominal abscess	Percutaneous drainage
3a	Ascites	Percutaneous drainage
3a	Ascites	Percutaneous drainage
3a	Pleural fluid	Percutaneous drainage
3a	Pleural fluid	Percutaneous drainage
3a	Wound secretion/Ascites	Resuture of operative drain at bedside
3a	Wound infection with subcutaneous abscess	Open drainage at bedside
3a	Stenosis of superior mesenteric artery	Angiography and stenting
3a	Left sided pneumothorax	Chest tube
3b	Portal vein thrombosis after portal vein reconstruction<br>Wound dehiscence	Reoperation x 2
3b	Hepatic necrosis	Reoperation
4	Hemorrhage + Kidney failure	Reoperation
4	Ascites + Chyle leak + Kidney and liver failure	Percutaneous drainage +<br>Transjugular intrahepatic portosystemic shunt +<br>Embolization of splenic artery

Supplementary Table S2. Rate of involvement of individual margins of pancreatectomy specimens.

	All patients	Pancreatoduodenectomy (n=46)	Distal pancreatectomy (n=3)	Total pancreatectomy (n=5)
Margin status*				
R0	7 (13)	5	1	1
R1	46 (85.2)	40	2	4
R2¤	1 (1.9)	1	0	0
Bile duct margin				
R0	51	46	NA	5
R1		0		0
Proximal gastric/duodenal margin				
R0	51	46	NA	5
R1		0		0
Pancreatic neck margin				
R0	44	37	2	5
R1	10	9	1	0
Anterior surface				
R0	45	39	2	4
R1	9	7	1	1
Posterior surface				
R0	43	37	2	4
R1	11	9	1	1
Superior mesenteric vein margin				
R0	26	24	NA	2
R1	25	22		3
Superior mesenteric artery margin				
R0	31	27	NA	4
R1	19	18		1
R2	1	1		
Number of involved margins				
0	14	12	1	1
1	18	15	1	2
2	14	11	1	2
3	5	5	0	0
4	3	3	0	0
5	0	0	0	0
6	0	0	0	0
7	0	0	0	0

*One patient underwent surgery abroad and had missing data. ¤R2 based on the pathology report with one grossly positive resection margin.

Supplementary Table S3. Site of first recurrence.

	Overall (n=54)	Pancreato-duodenectomy (n=46)	Distal pancreatectomy (n=3)	Total pancreatectomy (n=5)
**Patients with recurrence**	39 (72.2)	34 (73.9)	1 (33.3)	4 (80)
**Site of first recurrence**				
Locoregional	11 (28.2)	11		
Distant	19 (48.7)	17	1	2
Locoregional + distant	9 (23.1)	7		2
**Distant sites***				
Liver	15 (38.5)	14		1
Lung	10 (25.6)	8	1	1
Peritoneal carcinomatosis	12 (30.8)	9		3
Lymph nodes	5 (12.8)	5		
Bone	1 (2.6)	1		

Values are expressed as n (%). *No. and % add to more than 100% due to some patients experiencing recurrence at multiple sites.

Supplementary Table S4. The estimated means with 95 % confidence intervals (CI) of EORTC QLQ-C30 subscales.

	Baseline<br>Mean, CI<br><br>n=44	Before surgery<br>after NAT<br>Mean, CI<br>n=43	6 weeks<br>postop.<br>Mean, CI<br>n=14	3 months<br>postop.<br>Mean, CI<br>n=29	6 months<br>postop.<br>Mean, CI<br>n=29	12 months<br>postop.<br>Mean, CI<br>n=10
Physical functioning	78.4<br>(72.3,84.6)	72.9<br>(66.6,79.1)	58.9<br>(49.3,68.6)	67.9<br>(60.7,75.1)	74.8<br>(67.6,82.0)	74.8<br>(63.8,85.8)
Role functioning	60.2<br>(51.3,69.1)	58.8<br>(49.7,67.9)	40.2<br>(26.9,53.5)	51.5<br>(41.3,61.7)	59.6<br>(49.4,69.8)	64.9<br>(49.5,80.4)
Emotional functioning	76.6<br>(70.4,82.8)	81.1<br>(74.7,87.4)	75.0<br>(64.9,85.1)	77.8<br>(70.5,85.2)	77.1<br>(69.7,84.4)	77.4<br>(65.3,89.4)
Cognitive functioning	82.7<br>(76.1,89.2)	81.8<br>(75.2,88.5)	75.8<br>(65.7,86.0)	77.9<br>(70.4,85.5)	80.9<br>(73.3,88.5)	85.5<br>(73.6,97.5)
Social functioning	66.6<br>(58.8,74.5)	60.7<br>(52.7,68.8)	55.4<br>(43.0,67.8)	53.7<br>(44.5,62.8)	56.5<br>(47.3,65.7)	68.5<br>(53.9,83.1)
Global quality of life	58.0<br>(51.0,65.1)	66.3<br>(59.0,73.6)	47.0<br>(35.9,58.0)	50.8<br>(42.6,59.0)	58.8<br>(50.5,67.0)	63.2<br>(50.2,76.2)
Fatigue	44.7<br>(37.2,52.3)	45.6<br>(37.9,53.3)	56.6<br>(45.1,68.2)	50.9<br>(42.3,59.6)	47.2<br>(38.5,55.9)	43.8<br>(30.2,57.3)
Nausea and vomiting	14.0<br>(8.8,19.2)	12.6<br>(7.3,18.0)	20.7<br>(12.0,29.3)	12.8<br>(6.6,19.0)	13.1<br>(6.9,19.3)	9.3<br>(-1.1,19.7)
Pain	33.1<br>(25.2,41.0)	18.5<br>(10.4,26.6)	43.2<br>(30.4,56.1)	30.2<br>(20.9,39.6)	26.9<br>(17.6,36.3)	23.9<br>(8.6,39.2)
Dyspnoea	19.5<br>(11.7,27.3)	24.8<br>(16.8,32.7)	31.8<br>(20.1,43.6)	24.8<br>(15.8,33.7)	18.2<br>(9.2,27.1)	11.8<br>(-1.9,25.5)
Insomnia	40.6<br>(31.6,49.5)	27.6<br>(18.4,36.8)	51.0<br>(36.6,65.5)	32.7<br>(22.1,43.3)	35.9<br>(25.3,46.5)	28.6<br>(11.5,45.8)
Appetite loss	36.8<br>(27.2,46.3)	31.2<br>(21.4,41.0)	58.6<br>(42.7,74.5)	38.3<br>(26.9,49.7)	29.4<br>(17.8,41.0)	20.7<br>(1.6,39.8)
Constipation	20.0<br>(12.5,27.4)	21.4<br>(13.8,29.1)	13.7<br>(0.5,26.8)	20.5<br>(11.4,29.7)	16.2<br>(7.0,25.3)	10.2<br>(-5.9,26.2)
Diarrhoea	28.5<br>(19.2,37.8)	23.0<br>(13.4,32.6)	51.3<br>(35.5,67.0)	41.0<br>(29.8,52.2)	47.2<br>(36.0,58.5)	41.4<br>(22.4,60.4)
Financial difficulties	8.4<br>(1.3,15.6)	12.6<br>(5.4,19.9)	14.6<br>(5.2,24.0)	13.7<br>(6.0,21.5)	16.6<br>(8.8,24.4)	8.5<br>(-2.0,19.0)
